# Higher affinities of fibers with cell receptors increase the infection capacity and virulence of human adenovirus type 7 and type 55 compared to type 3

**DOI:** 10.1128/spectrum.01090-23

**Published:** 2023-11-29

**Authors:** Qiong Zhang, Zhichao Zhou, Ye Fan, Tiantian Liu, Yubing Guo, Xiao Li, Wenkuan Liu, Liling Zhou, Yujie Yang, Chuncong Mo, Yong Chen, Xiaohong Liao, Rong Zhou, Zhenhua Ding, Xingui Tian

**Affiliations:** 1 State Key Laboratory of Respiratory Disease, National Clinical Research Center for Respiratory Disease, Guangzhou Institute of Respiratory Health, The First Affiliated Hospital of Guangzhou Medical University, Guangzhou Medical University, Guangzhou, China; 2 Guangzhou Laboratory, Guangzhou, China; 3 Department of Radiation Medicine, Guangdong Provincial Key Laboratory of Tropical Disease Research, School of Public Health, Southern Medical University, Guangzhou, China; 4 School of Public Health, Guangdong Pharmaceutical University, Guangzhou, China; Hospital Saint-Louis, Paris, France

**Keywords:** respiratory viruses, adenoviruses, receptors, pneumonia, virulence, pathogenesis

## Abstract

Human adenovirus (HAdV) type 7 and type 55 infections cause more severe pneumonia than type 3 infections. However, the underlying mechanisms remain unclear. This study provided evidence linking the receptor-binding protein, fiber, to the stronger virulence of HAdV-55 and HAdV-7. Wild-type HAdV-7 and HAdV-55 produced larger plaques and grew more efficiently, resulting in a higher viral yield than HAdV-3. The recombinant knobs of HAdV-55 and HAdV-7 had higher binding affinities with desmoglein 2 (DSG2) than the HAdV-3 knob. Fiber-chimeric rAdV3E-K7 and rAdV3E-K55 were generated by replacing the knob of recombinant HAdV-3 (rAd3E) with knobs from HAdV-7 and HAdV-55, respectively. Full-length fiber-replaced rAd5-F3, rAd5-F7, and rAd5-F55 were generated from replication-defective HAdV-5. The replication and infectivity of these adenoviruses were compared using passage and primary cell and DSG2-humanized mouse models. Both rAdV3E-K7 and rAdV3E-K55 produced larger plaques and adsorbed more efficiently than rAd3E in A549 cells and primary human bronchial epithelial cells. Additionally, rAd5-F7 and rAd5-F55 showed stronger infectivity than rAd5-F3. Finally, rAdV3E-K7 and rAdV3E-K55 infections contributed to slightly more but not significant severe pneumonia than rAd3E in the humanized mouse model. In summary, higher binding affinities of fibers with DSG2 lead to higher infection efficiency of HAdV-7 and HAdV-55 compared to HAdV-3. This study provides new insights into the mechanisms of differential pathogenicity between HAdV types. It may have implications for the surveillance of highly pathogenic adenovirus strains and the development of novel adenovirus vectors and vaccines.

**IMPORTANCE:**

HAdV-3, -7, and -55 are the predominant types causing acute respiratory disease outbreaks and can lead to severe and fatal pneumonia in children and adults. In recent years, emerging or re-emerging strains of HAdV-7 and HAdV-55 have caused multiple outbreaks globally in both civilian and military populations, drawing increased attention. Clinical studies have reported that HAdV-7 and HAdV-55 cause more severe pneumonia than HAdV-3. This study aimed to investigate the mechanisms explaining the higher severity of HAdV-7 and HAdV-55 infection compared to HAdV-3 infection. Our findings provided evidence linking the receptor-binding protein fiber to stronger infectivity of the strains mentioned above by comparing several fiber-chimeric or fiber-replaced adenoviruses. Our study improves our understanding of adenovirus infection and highlights potential implications, including in novel vector and vaccine development.

## INTRODUCTION

Human adenovirus (HAdV) is a non-enveloped, double-stranded DNA virus with a genome of approximately 35 Kbp (1, 2). To date, 113 HAdV genotypes from 7 species (A–G) have been reported (http://hadvwg.gmu.edu/). HAdVs cause acute respiratory disease (ARD), gastroenteritis, hemorrhagic cystitis, and keratoconjunctivitis. Human adenoviruses usually cause mild, self-limiting infections in immunocompetent individuals (3 – 5). However, the recent World Health Organization report highlights human adenovirus as the possible etiology of unexplained infectious hepatitis (6, 7). HAdVs account for about 4% to 10% of community-acquired pneumonia (8). Severe adenovirus pneumonia can be complicated by respiratory failure, acute respiratory distress syndrome, encephalitis, sepsis, myocarditis, and even death (6, 8 – 10). Some adenovirus characteristics suggest the potential risk for a pandemic (11). Many patients develop permanent lung damage with sequelae such as bronchiectasis, bronchiolitis obliterans, and Swyer-James syndrome.

Of the HAdVs relevant to ARD, HAdV-3, -7, -14, and -55 of species B are the major types leading to ARD outbreaks and have caused severe and even fatal pneumonia in children and adults (12 – 16). HAdV-3 and -7 are the two predominant types causing ARD in children, and HAdV-7d has recently re-emerged in Asia and the United States, resulting in fatal pneumonia outbreaks (15, 17). The two emerging types, HAdV-14 and -55, have led to numerous outbreaks worldwide in civilian and military populations since 2006. HAdV-55 is a common pathogen among pediatric patients with severe pneumonia in China (18). It is an intertypic recombinant of HAdV-11 and HAdV-14, sharing a similar fiber protein with HAdV-14 (18, 19). The only approved live oral vaccine comprising HAdV types 4 and 7 has been used in the US military for 40 years but has not been approved for use in the general population in any part of the world (20).

Some clinical reports indicate that HAdV-7 and HAdV-55 infections cause more severe disease than type 3. Our recent study found that the severe illness rate was significantly higher in pediatric patients with HAdV-7 (51.4%, 36/70) than in those with HAdV-3 (19.7%, 14/71) and other types of HAdV (20%, 2/10) (21). In another molecular epidemiology study between 2010 and 2021, of the 29 deaths in the ICU, 27 were HAdV-7 positive, and the remaining 2 were HAdV-3 and HAdV-55 (22). Other reports have also suggested HAdV-7 causes more severe pneumonia and higher mortality in children than HAdV-3 (14, 15, 23 – 25). Case-control studies have found that HAdV-55 causes the same or more severe disease than HAdV-7, resulting in multiple fatal infections across China (18, 26, 27). However, the reason why HAdV-7 and HAdV-55 are more likely to cause life-threatening infections than HAdV-3 remains unknown (28, 29).

Viral receptors play key roles in viral infection, transmission, and pathogenesis. Recognized receptors may contribute to HAdV tissue tropism. The adsorption of adenovirus particles to receptors on respiratory mucosal cells is a key step in viral infection. High-affinity binding of the viral trimeric fiber protein to a cell surface primary receptor is a common feature shared by all adenovirus serotypes and is the first step in the infection of cells. The fiber’s C-terminal globular domain node (knob) binds to host cell receptors that mediate adenovirus infection. The coxsackie-adenovirus receptor is the main adsorption receptor for most adenoviruses, except for B species. HAdV-3, -7, -11, and -14 use the epithelial junction protein desmoglein 2 (DSG2) as a primary receptor for infection (30 – 32). Feng et al. also found that DSG2 played a major role in mediating HAdV-55 infection (32). HAdVs targeting DSG2 may lead to airway epithelial barrier dysfunction (33, 34). Whole-genome comparison analysis indicated that the difference between HAdV-3 and HAdV-7 mainly occurred in fiber proteins (35, 36). Therefore, we hypothesized that HAdV-7 and -55, which cause more severe diseases than type 3, may be associated with high-affinity binding between the trimeric fiber protein and the DSG2 receptor. However, no report has provided evidence for the association of HAdV-3, HAdV-7, and HAdV-55 fibers with virulence.

In this study, we constructed a series of recombinant chimeric adenoviruses with fiber replacement to investigate the role of fibers in HAdV-7 and HAdV-55, which are associated with stronger infectivity and virulence compared to HAdV-3, using cell models and a humanized DSG2 mouse model.

## RESULTS

### Infection and replication of wild HAdV-55, HAdV-7, and HAdV-3 strains

**Fig 1:**
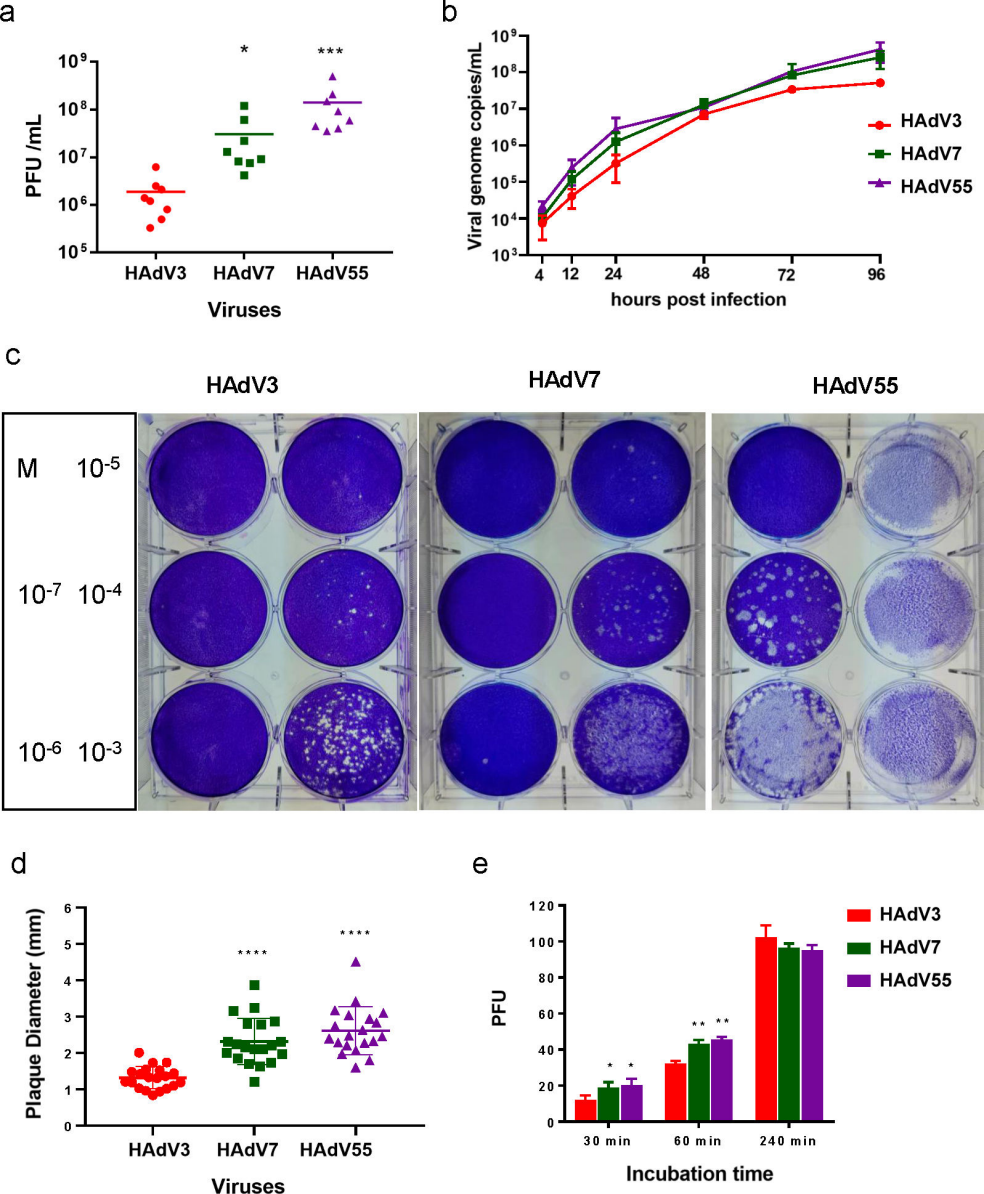
Infection and replication characterization of wild HAdV-3, HAdV-7, and HAdV-55. (a) Plaque formation of HAdV-3, HAdV-7, and HAdV-55 clinical isolates. Each symbol represents an individual isolate of the virus. (b) Viral genome of HAdV-3, HAdV-7, and HAdV-55. HAdVs in virus-infected A549 cells were determined at 4, 12, 24, 48, 72, and 96 h post-infection using qPCR. Plaque formation (c) and size distribution (d) of wild HAdV-3, HAdV-7, and HAdV-55. (e) A549 cells were infected with HAdV-3, HAdV-7, and HAdV-55 for 30, 60, or 240 min, washed three times, and the plaque formation units were detected. Each experiment was repeated three times independently, and the mean values and standard deviations were shown. Statistical analysis was performed using the Kruskal-Wallis test, followed by Dunn’s multiple comparisons test. ****P < 0.0001; ***P < 0.001.

Clinical data indicated that HAdV-55 and HAdV-7 caused more severe disease than HAdV-3. Here, we compared viral infection and replication of these three HAdV types *in vitro*. We compared the viral titers of clinically isolated HAdV-3, -7, and -55 strains. The mean viral titers of HAdV-55 and HAdV-7 isolates were significantly higher than those of HAdV-3 (Fig. 1a) (*P* < 0.001). This result shows that Ad7 and Ad55 could proliferate better *in vitro* than Ad3. HAdV-3, HAdV-7, and HAdV-55 growth kinetics were detected at 4, 12, 24, 48, 72, and 96 h post-infection. HAdV-7 and HAdV-55 grew faster than HAdV-3 (Fig. 1b). Plaque assays were performed to compare plaque number and size (Fig. 1c). The plaque diameters of HAdV-3, HAdV-7, and HAdV-55 were 1.32 ± 0.30 mm, 2.31 ± 0.64 mm, and 2.61 ± 0.65 mm, respectively. The plaque size of HAdV-3 was significantly smaller than those of HAdV-7 and HAdV-55 (*P* < 0.0001) (Fig. 1d). Bigger plaque sizes of HAdV-7 and HAdV-55 may reflect stronger virulence *in vitro*. The viruses were used to infect A549 cells with the same titers for 30, 60, or 240 min, and the cells were then washed and cultured for 9 days to detect plaque formation units. More plaque-forming units (PFUs) were present in cells infected with HAdV-55 and HAdV-7 than in cells infected with HAdV-3 after adsorbing for 30 or 60 min but not 240 min (Fig. 1e). This result showed that HAdV-7 and HAdV-55 adsorb cells faster than HAdV-3. These results demonstrate that HAdV-7 and HAdV-55 had higher infection and replication efficiencies than HAdV-3.

### Infection and replication of recombinant rAdV55E, rAdV7E, and rAdV3E

In further experiments, recombinant replication-competent Ads expressing enhanced green fluorescent protein (EGFP) were used for analysis, and infection and replication characteristics of rAdV55E, rAdV7E, and rAdV3E were compared. The plaque diameters of rAdV3E, rAdV7E, and rAdV55E were 1.97 ± 0.86 mm, 3.36 ± 1.05 mm, and 2.65 ± 0.37 mm, respectively. The plaque size of rAdV3E was significantly smaller than those of rAdV7E and rAdV55E (*P* < 0.01) (Fig. S1a). rAdV7E and rAdV55E viral titers were significantly higher than those of rAdV3E (Fig. S1b) (*P* < 0.05). The recombinant viruses were used to infect A549 cells with the same viral genome copies for 30 or 60 min, and the cells were then washed and cultured for 24 h. Green fluorescent cells were observed and counted. More fluorescent cells were present in cells infected with rAdV55E and rAdV7E than in cells infected with rAdV3E for 60 min (Figure S1c and d). The same genome copies of rAdV3E, rAdV7E, or rAdV55E were used to infect cells, and viral titers were determined at 4, 12, 24, 48, 72, and 96 hpi to analyze growth kinetics. Viral titer was determined using qPCR to quantify viral genome copies and fluorescence cell count to detect fluorescence-forming units (FFU). rAdV7E and rAdV55E had higher viral genome copies than rAdV3E after 4 hpi (Fig. S1e) and grew to higher FFUs than rAdV3E after 24 hpi (Fig. S1f). These results demonstrate that rAdV7E and rAdV55E have higher infectivity and replication efficiency than rAdV3E.

### HAdV-7 and HAdV-55 fiber knobs exhibited higher affinity with DSG2 than the HAdV-3 fiber knob

**Fig 2:**
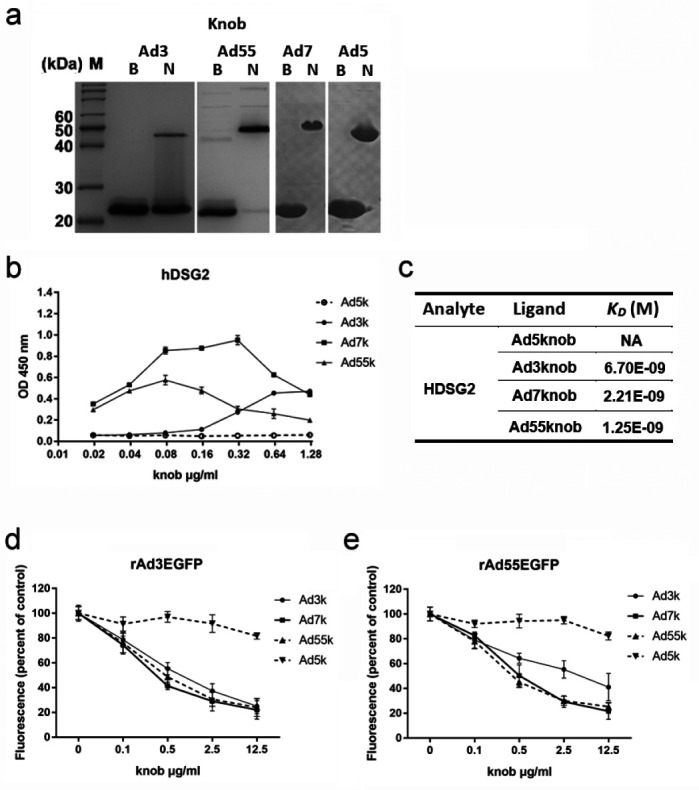
Affinity analysis of receptor DSG2 binding fiber knobs of HAdV-3, HAdV-7, and HAdV-55. (a) SDS-PAGE of the purified recombinant fiber knobs of HAdV-3, HAdV-7, HAdV-55, and HAdV-5. Purified protein in loading buffer was incubated at room temperature for 5 min and then incubated on ice (native, N) or denatured at 98°C for 5 min (boiled, B). The image labeled “Knob: Ad3” is identical to the image labeled “HAdV-B3-Knob” in our previously published article (37). The Ad3-knob protein used in both articles is the same one prepared from the same batch. (b) Comparison of the affinities of knobs binding DSG2 by ELISA. The plates were coated with 1.5 µg/mL DSG2 in HBS-N (Ca^2+^) and then incubated with serially diluted Ad3K, Ad7K, Ad55K, and Ad5K. Afterward, the HRP-labeled anti-His antibody was added. Finally, the substrate was added to read A450. (c) Affinity measurement and comparison of receptor DSG2 binding with fiber knobs of HAdV-3, HAdV-7, and HAdV-55 by SPR. (d and e) Knob competition experiments. Serially diluted recombinant knobs in phosphate-buffered saline (PBS) were added to A549 cells and incubated on ice. Subsequently, EGFP-expression HAdV rAd3EGFP (d) or rAd55EGFP (e) was added. After washing twice with cold PBS, the cells were cultured in a fresh medium for 2 days. Cells were photographed, and the number of fluorescent cells was determined. HAdV-5 fiber knob was used as the negative control.

HAdV-3 and HAdV-7 infect epithelial cells through fiber knob binding to the cellular receptor human desmoglein-2. Human desmoglein-2 also plays a major role in infection with HAdV type 55. The fiber knobs of HAdV-7, -55, and -5 were expressed and purified as trimeric forms in PBS; however, a part of the HAdV-3 fiber knob appears as a monomer (Fig. 2a). The knobs and receptor DSG2 affinities were measured and compared by ELISA and surface plasmon resonance (SPR). As expected, the control HAdV-5 knob (Ad5k) did not bind DSG2. The affinity of Ad55k for DSG2 (*K_D_* = 1.25 × 10^−9^ M) was slightly higher than that of Ad7k (*K_D_* = 2.21 × 10^−9^ M) and markedly higher than that of Ad3k (*K_D_* = 6.70 × 10^−9^ M) (Fig. 2b and c). Ad7k and Ad55k displayed stronger inhibition efficiency against recombinant rAd3EGFP and rAd55EGFP (Fig. 2d and e). These results indicated that HAdV-7 and HAdV-55 fiber knobs have a higher affinity for DSG2 than the HAdV-3 fiber knob.

### Infection and replication of rAdV3E-K7, rAdV3E-K55, and rAdV3E

**Fig 3:**
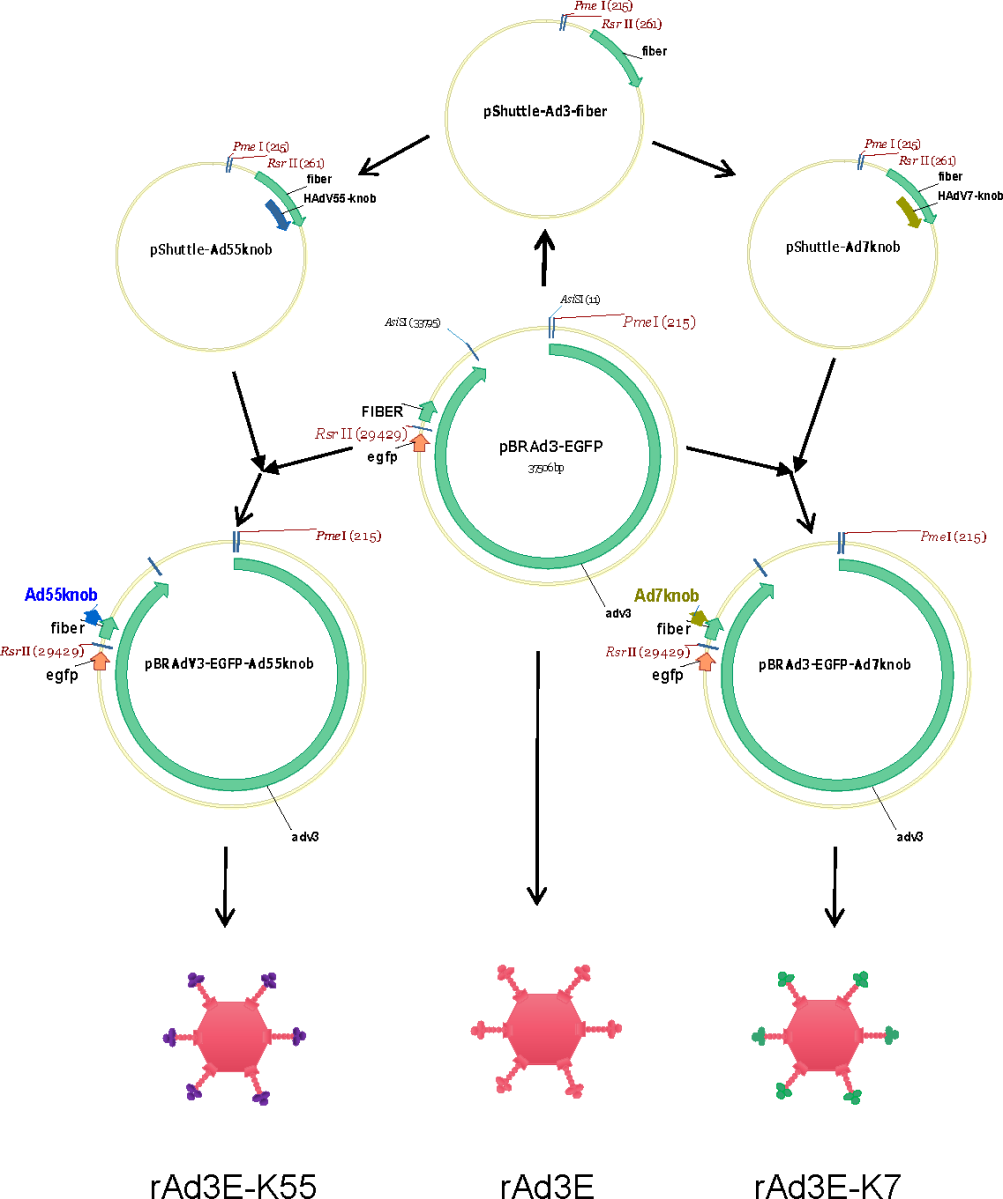
Sketch of recombinant replication competent and fiber-chimeric rAd3E-K7 and rAdV3E-K55 preparation. rAd3E-K7 and rAd3E-K55 possess the same skeleton as rAd3E and a chimeric fiber with a replaced knob from HAdV-7 (green marked) and HAdV-55 (purple marked), respectively.

**Fig 4:**
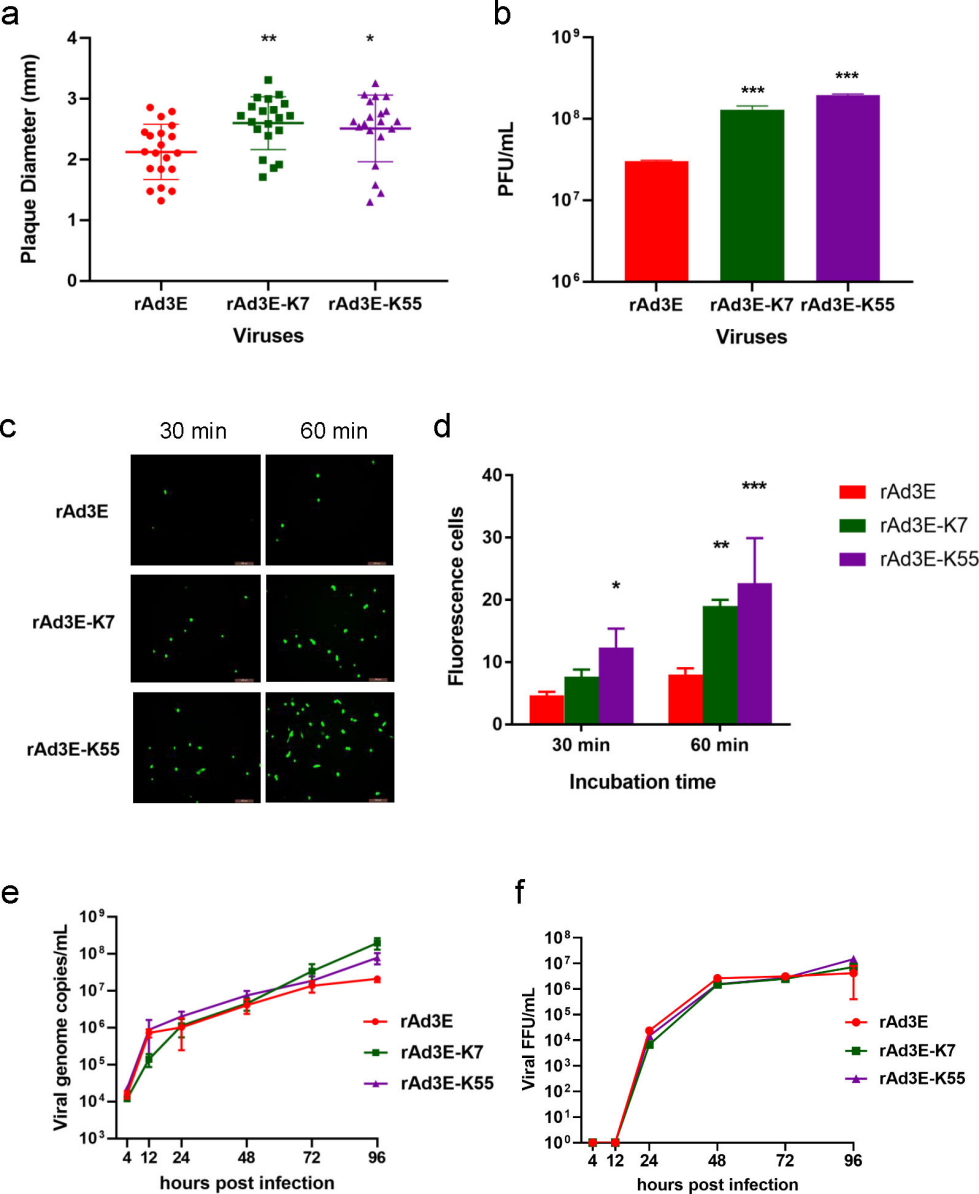
Infection and replication characterization of recombinant rAd3E, rAd3E-K7, and rAd3E-K55. Plaque size distribution (a) and plaque formation unit (b) of rAd3E, rAd3E-K7, and rAd3E-K55. (c) A549 cells were infected with rAd3E, rAd3E-K7, and rAd3E-K55 for 30 or 60 min, washed three times, and observed under a fluorescence microscope at 24 h post-infection. (d) Fluorescent cell numbers were counted 24 h post-infection. (e) Viral genome DNA proliferation curves and (f) infectious virus growth kinetics of rAd3E, rAd3E-K7, and rAd3E-K55. Viral genome copy numbers were determined by qPCR. Infectious virus titers were determined by counting fluorescence-forming units. The viruses infected in A549 cells were collected and determined at 4, 12, 24, 48, 72, and 96 h post-infection. Each experiment was repeated three times independently, and the mean values and standard deviations were shown. Statistical analysis was performed using the Kruskal-Wallis test, followed by Dunn’s multiple comparisons tests. *P* < 0.001; *P* < 0.01; *P* < 0.05.

To explore the role of the knob in viral infection and exclude the influence of other viral proteins such as hexon, two recombinant replicative knob-replaced adenoviruses, expressing EGFP, rAd3E-K55, and rAd3E-K7, were obtained by replacing the fiber knob region of rAd3E with the fiber knob region of HAdV-55 and HAdV-7, respectively (Fig. 3). These two chimeric viruses were then used to compare infection and replication characteristics with rAd3E. The plaque diameters of rAdV3E, rAdV3E-K7, and rAdV3E-K55 were 2.30 ± 0.54 mm, 2.60 ± 0.44 mm, and 2.44 ± 0.61 mm, respectively. The plaque size of rAdV3E was smaller than those of rAdV3E-K7 and rAdV3E-K55 (*P* < 0.05) (Fig. 4a). The viral plaque titers of rAdV3E-K7 and rAdV3E-K55 were significantly higher than that of rAdV3E (Fig. 4b) (*P* < 0.05). The recombinant viruses were used to infect A549 cells for 30 or 60 min, and cells were then washed and cultured for 24 h. rAdV3E-K7 and rAdV3E-K55 produced more fluorescent cells than rAdV3E after 30 or 60 min of incubation, demonstrating that rAdV3E-K7 and rAdV3E-K55 adsorbed cells more efficiently than rAd3E (Fig. 4c and d). The rAdV3E, rAdV3E-K7, and rAdV3E-K55 were used to infect cells, and viral titers were determined at 4, 12, 24, 48, 72, and 96 hpi to analyze growth kinetics. However, rAdV3E-K7 and rAdV3E-K55 had higher genome copies than rAdV3E only at 96 hpi (Fig. 4e) and grew to similar FFUs at different times post-infection (Fig. 4f). The rAdV3E, rAdV3E-K7, and rAdV3E-K55 showed similar genomic DNA replication efficiency. These results demonstrate that rAdV3E-K7 and rAdV3E-K55 had greater infectivity but not replication efficiency than rAdV3E in A549 cells.

### Infection and replication of rAdV5-F7, rAdV5-F55, and rAdV5-F3

**Fig 5:**
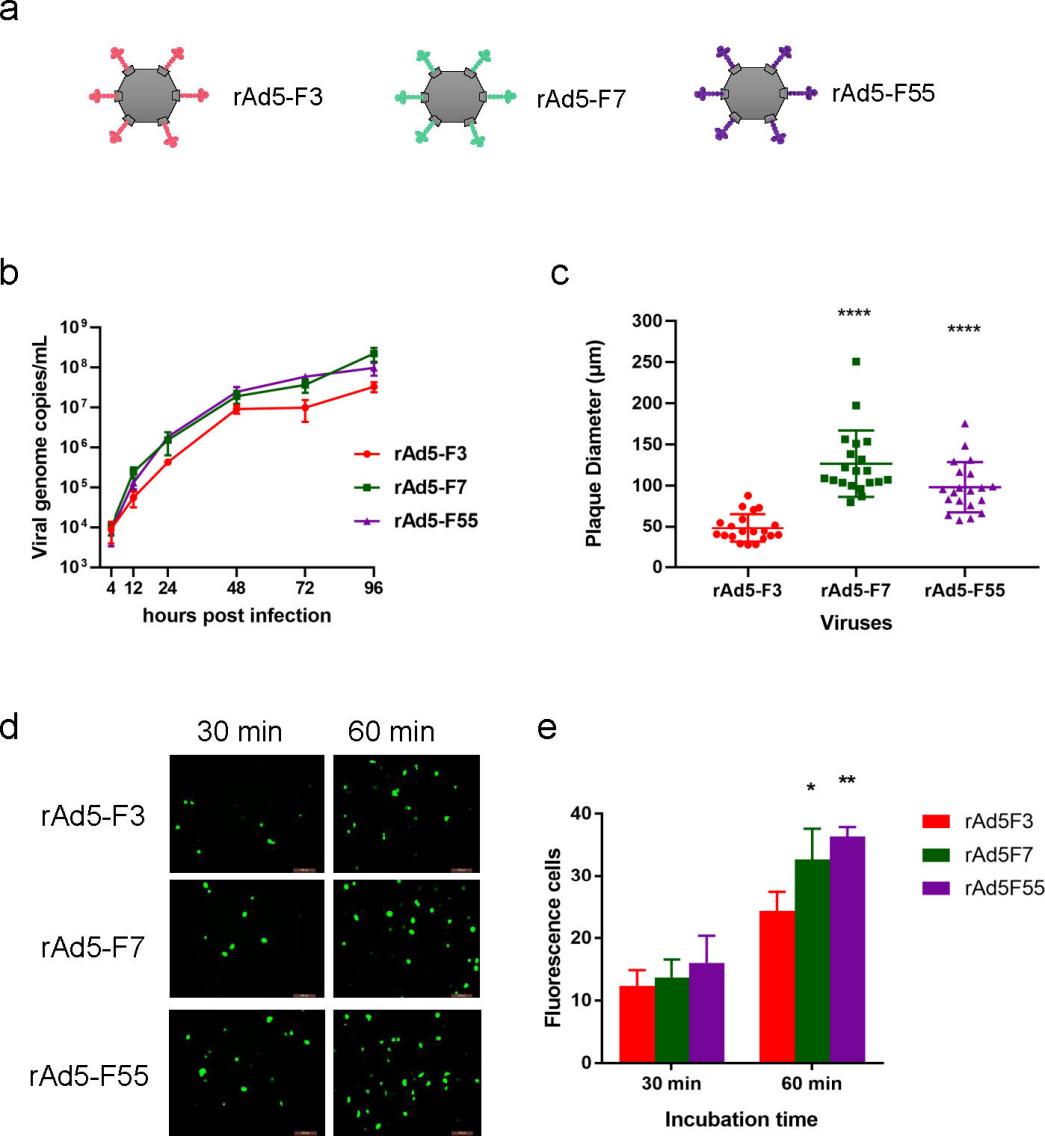
Infection and replication characterization of recombinant rAd5-F3, rAd5-F7, and rAd5-F55. (a) Sketch of recombinant replication-deficient and fiber-replaced chimeric adenovirus type 5. rAd5-F3, rAd5-F7, and rAd5-F55 possess the same skeleton from rAd5 and replaced fiber from HAdV-3 (red marked), HAdV-7 (green marked), and HAdV-55 (purple marked), respectively. (b) Viral genome DNA proliferation curve kinetics of rAd5-F3, rAd5-F7, and rAd5-F55. Viral genome copy numbers were determined using qPCR. The viral particles in infected 293T cells were collected and measured at 4, 12, 24, 48, 72, and 96 h post-infection. (c) Plaque size distribution of rAd5-F3, rAd5-F7, and rAd5-F55. (d) 293T cells were infected with rAd5-F3, rAd5-F7, and rAd5-F55 for 30 or 60 min, washed three times, and observed under a fluorescence microscope at 24 h post-infection. Fluorescent cell numbers were counted. (e) Each experiment was repeated three times independently, and the mean values and standard deviations were shown. Statistical analysis was performed using the Kruskal-Wallis test, followed by Dunn’s multiple comparisons tests. *P* < 0.0001; *P* < 0.01; *P* < 0.05.

We prepared three recombinant replication-deficient HAdV-5 expressing EGFP, rAdV5-F55, rAdV5-F7, and rAdV5-F3, by replacing the full-length fiber region of rAd5E with the fibers of HAdV-55, HAdV-7, and HAdV-3, respectively (Fig. 5a). Heterologous fibers were properly incorporated into the Ad5 virions by western blot for the purified recombinant adenoviruses (data not shown). The fluorescence plaques of rAdV5-F3 were smaller than those of rAdV5-F7 and rAdV5-F55 (*P* < 0.05) (Fig. 5b). The viral plaque titers of rAdV5-F7 and rAdV5-F55 were significantly higher than those of rAdV5-F3. rAdV5-F7 and rAdV5-F55 replicated faster and generated more virus copies than rAdV5-F3 (Fig. 5c). 293T cells were infected with the recombinant viruses for 30 or 60 min of incubation and then washed and cultured for 24 h. rAdV5-F7 and rAdV5-F55 produced more fluorescent cells than rAdV5-F3 (Fig. 5d and e). These results showed that rAdV5-F7 and rAdV5-F55 had stronger infectivity than rAdV5-F3.

### Infection and replication of adenoviruses in HBEpiCs

**Fig 6:**
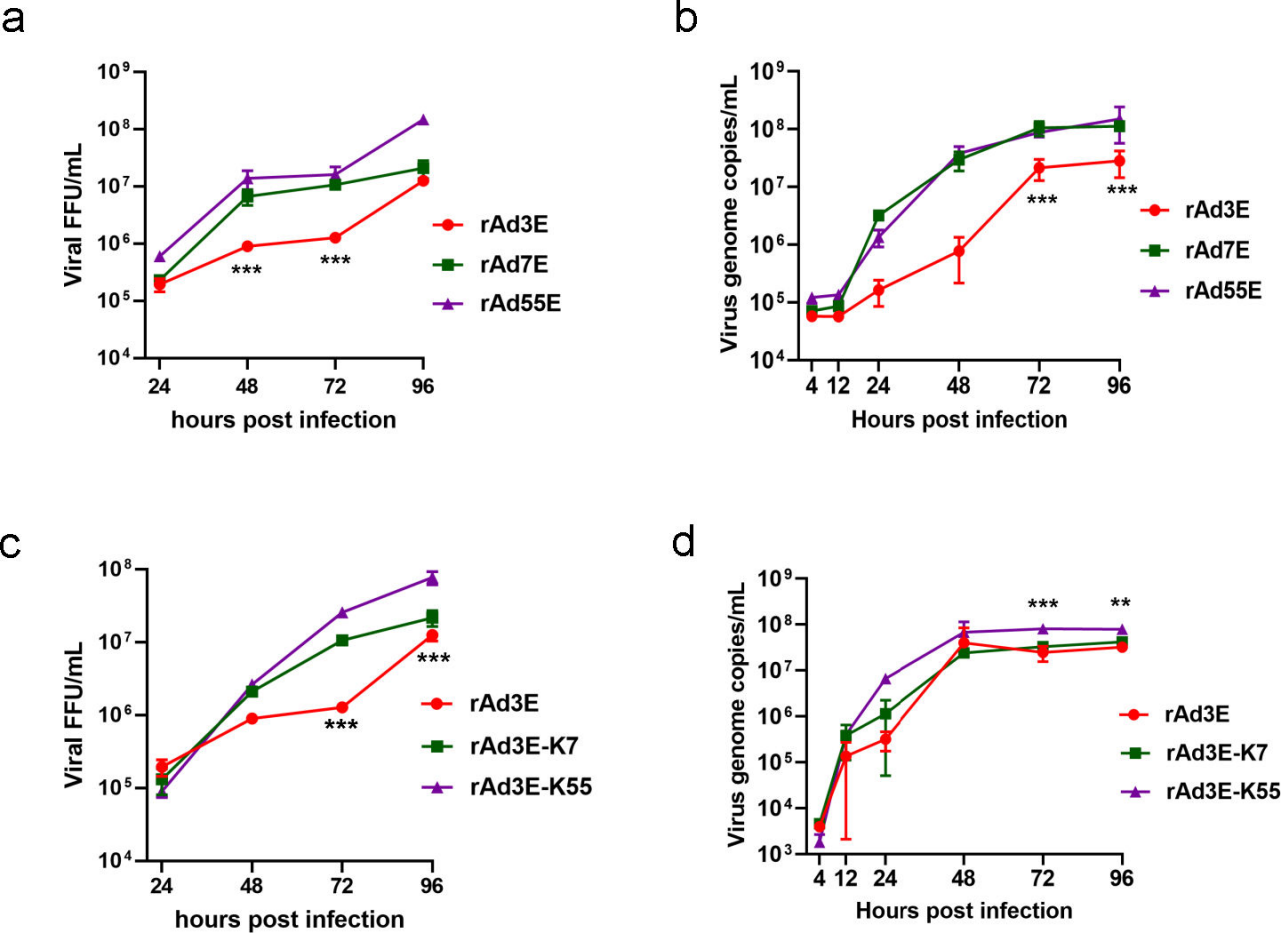
Infection and replication characterization of rAdV3E, rAdV7E, rAdV55E, rAdV3E-K7, and rAdV3E-K55 in HBEpiCs. (a) Infectious virus growth kinetics and (b) viral genome DNA proliferation curves of rAdV3E, rAdV7E, and rAdV55E. (c) Infectious virus growth kinetics and (d) viral genome DNA proliferation curves of rAdV3E, rAdV3E-K7, and rAdV3E-K55. Viral genome copy numbers were determined by qPCR. Infectious virus titers were determined by counting FFU. The viral particles in infected HBEpiCs were collected and quantified at 4, 12, 24, 48, 72, and 96 h post-infection. Each experiment was repeated three times independently, and the mean values and standard deviations were shown. Statistical analysis was performed using the Kruskal-Wallis test, followed by Dunn’s multiple comparisons tests. The two-way ANOVA with Dunnett’s multiple comarisons test was used to compare the means of groups at different times.

Human adenoviruses infect and replicate in respiratory tract cells. In undifferentiated Human bronchial epithelial cells (HBEpiCs), rAdV7E and rAdV55E replicated to produce significantly higher FFUs (Fig. 6a) and genome copies (Fig. 6b) than rAdV3E after 24 hpi. The knob-replaced adenoviruses, rAdV3E-K7 and rAdV3E-K55, also replicated and produced significantly higher FFU of viruses at 48 and 72 hpi than rAdV3E (Fig. 6c) (*P* < 0.001); rAdV3E-K55 replicated to produce significantly higher genome copies of viruses than rAdV3E at 72 and 96 hpi (Fig. 6d). These results showed that rAdV3E-F7 and rAdV3E-F55 had stronger replication efficiency than rAdV3E in HBEpiCs.

The differentiated HBEpiC model was established and infected with replicable adenoviruses rAdV3E, rAdV7E, rAdV55E, rAdV3E-K7, and rAdV3E-K55. rAdV7E, rAdV55E, rAdV3E-K7, and rAdV3E-K55 produced more fluorescent cells and larger fluorescent foci than rAdV3E (Fig. S2). The viral copies of rAdV7E, rAdV55E, rAdV3E-K7, and rAdV3E-K55 in the basolateral chambers were slightly but not significantly higher than those of rAdV3E after 6 days post-infection (dpi) (Fig. S3a and b). No virus was detected at 4 dpi. There was no significant difference in transepithelial electrical resistances (TEERs) of the differentiated HBEpiC models infected with these adenoviruses at 4 dpi (Fig. S3a and b). The TEERs of models infected with rAdV7E, rAdV55E, rAdV3E-K7, and rAdV3E-K55 fell faster than rAdV3E after 6 dpi.

### Humanized DSG2 receptor knock-in mouse model infected with rAdV3E-K7, rAdV3E-K55, and rAdV3E

We generated a humanized receptor knock-in mouse model, Ho-hDSG2-C57. Primary kidney cells from wild-type C57 mice (wild-c57) and DSG2-knock-in (KI) mice (Ho-hDSG2-C57) were separated and infected with rAd5-F3, rAd5-F7, and rAd5-F55. These three adenoviruses produced more fluorescent cells in Ho-hDSG2-C57 cells than in wild-c57 cells (*P* < 0.05) (Fig. S4a), which demonstrated that hDSG2 knock-in enhanced the efficiency of human adenovirus infection. rAd5-F7 and rAd5-F55 propagated more fluorescent cells from Ho-hDSG2-C57 mice than rAd5-F3 (*P* < 0.05) (Fig. S4a and b).

**Fig 7:**
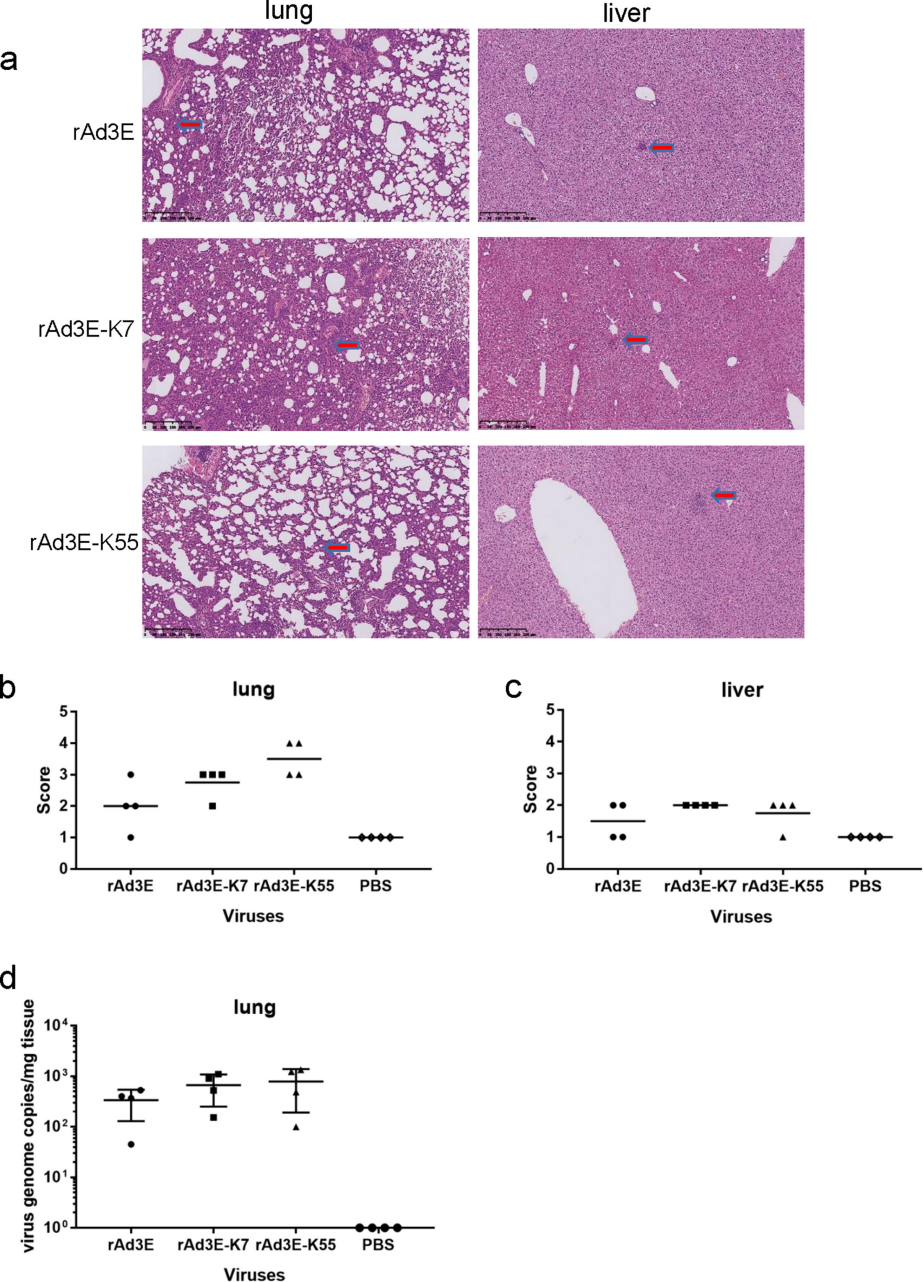
Humanized DSG2-KI mice intranasally infected with rAdV3E, rAdV3E-K7, and rAdV3E-K55. (a) Hematoxylin and eosin staining of lung and liver tissues. Arrows indicate pathological changes. (b) Lung injury scores and (c) liver injury scores. (d) Viral DNA load in lung tissues. Each symbol represents an individual mouse, and the horizontal lines indicate mean values or mean ± standard deviation. No significant differences were found (*P* > 0.05).

Ho-hDSG2-C57 mice were intranasally infected with rAd3E, rAd3E-K7, or rAd3E-K55. The lung tissues of infected mice mainly showed interstitial pneumonia, with significantly widened alveolar septa in some areas, vascular congestion, more acute and chronic inflammatory cell infiltration in local areas, and alveolar expansion and fusion. Overall severity was mild to severe (Fig. 7a). Histopathological changes in the liver demonstrated that adenoviruses produced relatively small liver shadows and focal infections (Fig. 7a). Lung injury scores showed that mice infected with rAd3E-K7 and rAd3E-K55 exhibited more but not significant severe pneumonia than rAd3E mice (Fig. 7b). Most mice infected with the adenovirus also showed mild liver injury (Fig. 7c). Detectable viral genome copies were slightly high but not significant in lung tissues of mice infected with rAd3E-K7 (668.62 ± 417.42) and rAd3E-K55 (789.00 ± 597.06) than that infected with rAd3E (335.75 ± 205.92) (Fig. 7d).

## DISCUSSION

Some clinical studies have indicated that HAdV-7 and HAdV-55 cause more severe diseases than type 3 (14, 15, 23 – 25). However, little is known regarding this mechanism. This study provided evidence linking the receptor-binding protein, fiber, to the stronger infectivity and virulence of HAdV-55 and HAdV-7.

Wild-type HAdV-7 and HAdV-55 produced larger plaques, showed higher infection abilities, and grew more efficiently than HAdV-3 (Fig. 1). Similar results were obtained for recombinant HAdVs expressing EGFP, rAd3E, rAd7E, and rAd55E (Fig. S1). These results suggest that HAdV-7 and HAdV-55 have higher infection abilities, possibly contributing to their stronger invasion capability than HAdV-3. Previous studies have indicated that DSG2 is the primary cell receptor for HAdV-7, HAdV-55, and HAdV-3 infection (30 – 32).

We found that the HAdV-55 and HAdV-7 fiber knobs had higher binding affinities with DSG2 than the HAdV-3 fiber knobs. Although a large part of the purified HAdV-3 fiber knob appears as a monomer, which may impact the results of the affinity test, the purified HAdV-3 fiber knob has the complete function of inhibiting HAdV-3 or HAdV-55 infection. The fiber shaft is important for the trimerization of fiber, and the entire fiber of HAdV-3 in adenovirus virions appears to be trimerized. Furthermore, we obtained two kinds of fiber-chimeric HAdVs, one replacing the receptor-binding domain (fiber knob) of rAd3E and another replacing the entire fiber of Ad5-based vector. rAdV3E-K7 and rAdV3E-K55 produced larger plaques and adsorbed more efficiently than rAd3E (Fig. 4); rAd5-F7 and rAd5-F55 also produced larger plaques and adsorbed more efficiently than rAd5-F3 (Fig. 5). The results demonstrated that higher affinities of fiber knobs with cell receptors increase the infection capacity of HAdV-55 and HAdV-7 compared to HAdV-3. It can be speculated that greater infectivity causes the virus to infect airway cells more efficiently.

Our results also partially demonstrate that higher affinities of fiber knobs with cell receptors may increase the virulence of HAdV-55 and HAdV-7 compared to HAdV-3. First, viral plaque size is often used to characterize viral virulence at cell levels. The larger plaques of rAdV3E-K7 and rAdV3E-K55 compared to rAd3E, rAd5-F7 and rAd5-F55 compared to rAd5-F3 indicated stronger virulence in cells. Inflammatory cytokines, such as IL-6, IL-8, and MCP-1 levels, were higher in human embryonic lung fibroblast cells (MRC-5) infected by rAdV3E-K7 and rAdV3E-K55 than those by rAdV3E both at 10 and 26 h post-infection (data not shown). Second, we also verified the hypothesis using primary HBEpiC models. The HBEpiC model could better simulate viral infection in the respiratory tract (38). rAd7E and rAd55E grew significantly more efficiently and had higher titers than rAd3E. rAdV3E-K7 and rAdV3E-K55 grew to a higher FFU at 72 hpi and generated higher genome copies at 48 hpi than rAdV3E. Differentiated HBEpiCs can form tight junctions to better simulate the respiratory epithelium *in vivo*, which contains four main cell types: surface epithelial, basal, goblet, and ciliated cells (38). In the differentiated HBEpiCs, rAdV3E-K7 and rAdV3E-K55 proliferated to larger fluorescent foci than rAdV3E (Fig. S2). TEERs of models of infected rAdV7E, rAdV55E, rAdV3E-K7, and rAdV3E-K55 collapsed faster compared to rAdV3E after 6 dpi, and higher titers of these viruses were observed in the basolateral chambers compared to rAdV3E (Fig. S3). TEER values and the virus copies in the basolateral chambers represent intercellular junctions. These results indicate that fibers of HAdV-7 and HAdV-55 may enhance the opening of intercellular junctions compared to those of HAdV-3. DSG2 is the primary high-affinity receptor used by HAdV-3, -7, and -55 and is a component of the apical junctional complex (AJC) (30, 32, 39). Adenoviral binding of DSG2 can trigger the opening of intercellular junctions, leading to disruption of the AJC and airway epithelial barrier dysfunction (40). Epithelial permeability may allow pathogens and inhaled allergens to invade subsequently (34). The binding of HAdV-7 and -55 to DSG2 with higher affinities may contribute to stronger AJC disruption than HAdV-3, allowing the virus to enter the blood. Chen et al. detected adenovirus DNA in serum samples from 40% and 4.2% of HAdV-7- and HAdV-3-infected children, respectively, and viremia was strongly associated with a severe clinical presentation (41). Therefore, this property may contribute to the stronger virulence of HAdV-7 and -55 than HAdV-3, which may lead to increased pathogenicity in humans.

Third, the DSG2-humanized mouse model was infected with rAdV3E, rAdV3E-K7, and rAdV3E-K55. This model was constructed using TALEN knock-in technology, in which the mouse DSG2 gene was silenced by the inserted human DSG2 gene. rAdV3E-K7 and rAdV3E-K55 infections contributed to higher pathological scores and higher viral DNA load in lung tissues than rAd3E, although the difference is not statistically significant. Although hDSG2 knock-in enhanced the efficiency of HAdV-B infection, the hDSG2 knock-in mouse is not yet a satisfactory model for HAdV-B infection. Previous studies have demonstrated that human adenoviruses cannot replicate in mice. This is a limitation of this study. Better animal models that support HAdV-B infection and replication should be developed for more detailed pathogenesis studies.

Our results demonstrated that fiber binding to cell receptors contributes to stronger infectivity and faster growth of HAdV-55 and HAdV-7 than HAdV-3. Stronger infectivity of the virus may lead to faster growth by spreading faster but not genome replication. Fiber might play a small role in the higher genome replication efficiency of HAdV-7 and HAdV-55 compared with HAdV-3. Besides fiber, there may be other mechanisms contributing to faster growth and stronger virulence of HAdV-55 and HAdV-7 compared to HAdV-3. The replication efficiency may also be associated with other virus genes, such as E1. Other viral factors might contribute to stronger virulence, such as the E1 gene and viral DNA polymerase affecting viral genome replication and E3 genes suppressing the cellular immune response (29), which should be investigated in further work.

Human adenoviruses evolve mainly through recombination. Highly pathogenic HAdV-55 is an intertypic recombinant of HAdV-11 and HAdV-14, sharing a similar fiber protein with HAdV-14. This study advocates that more attention should be paid to novel adenoviruses with a fiber protein with HAdV-7 or HAdV-55. This study also suggests that high-virulence HAdV may be attenuated by modifying fiber protein as a vaccine candidate.

### Conclusions

In summary, the results suggest that higher binding affinities of fibers with cell receptors may lead to higher infection efficiency which may be one of the key factors contributing to stronger virulence of HAdV-7 and HAdV-55 compared to HAdV-3. This study provides new insights into the mechanism underlying differences in clinical pathogenicity between HAdV-7, HAdV-55, and HAdV-3. It may also have implications for the surveillance of novel adenoviruses and the development of novel adenovirus vectors and vaccines.

## MATERIALS AND METHODS

### Virus strains and cells

Human lung adenocarcinoma 549 cells (A549) and 293T cells obtained from the ATCC were cultured in Dulbecco’s modified Eagle’s medium (DMEM) with 10% FBS and 1% penicillin/streptomycin (P/S). Human bronchial epithelial cells were cultured in keratinocyte serum-free medium plus 1 mM A83-01, 5 mM Y-27632, and 3 mM isoproterenol, which was dubbed epithelial expansion medium (EpiX) (42). HAdV-3 GZ1 (GenBank No. DQ099432.4) (43), HAdV-7 CQ1198 (GenBank No. JX625134), and HAdV-55 Shanxi-Y16 (GenBank No. MK123979) (18) strains were maintained in our laboratory, and the competent HAdV-3-based vector rAd3ΔE3GFP (rAd3E) expressing enhanced green fluorescent protein was generated as previously described and kept in our laboratory (44). Other HAdV strains were isolated from hospitalized children with severe ARD at Guangzhou Women and Children’s Medical Center or Beijing Children’s Hospital (18). All HAdVs were cultured in A549 cells or 293T cells and subsequently stored at −80°C in the State Key Laboratory of Respiratory Disease (Guangzhou, China), and all experiments were performed in the biological safety level-2 laboratory. HAdV particles were purified by standard cesium chloride gradient centrifugation and suspended in phosphate-buffered saline (PBS) (pH 7.4), as previously described (45).

### Affinity analysis between HAdV fiber knobs and receptor DSG2

Recombinant fiber knob peptides of HAdV-3, -5, -7, and -55, containing the last shaft repeat and an N-terminal His tag, Ad3K, Ad7K, Ad55K, and Ad5K, were expressed from the vector pQE30 in *E. coli* and purified using Ni-NTA His-Bind Resin (Novagen, EMD Millipore Corp., Billerica, MA, USA) under native conditions, as previously described (37, 46). The purified Ad knob peptides were stored at −80°C in our laboratory (37, 46). The purified recombinant peptides were subsequently mixed with 5× loading buffer and incubated on ice (native) or heated for 5 min at 98°C (boiled, B). The native or boiled samples were separated using 12% SDS-polyacrylamide gel electrophoresis.

The affinity of receptor DSG2 binding with fiber knobs of HAdV-3, -7, or -55 was measured by SPR. Briefly, recombinant human DSG2 (Creative Biomart, Shirley, NY, USA) was covalently immobilized onto the CM5 sensor chip via amine coupling chemistry under the following conditions: Surface activation on both flow cells 1 and 2 was achieved by injecting a freshly prepared “NHS + EDC” 1:1 mixture at a flow rate of 10 µL/min for 420 s; DSG2 (diluted in 10 mM sodium acetate, pH 4.5) was immobilized on flow cell 2 of channels 1–5 at a flow rate of 10 µL/min. Excess reactive groups were blocked with ethanolamine at a flow rate of 10 µL/min for 7 min. The immobilization level (RU) was 3,500–3,700. The assay was performed at 25°C using HBS-EP running buffer. A series of analyte concentrations were consecutively injected over the ligand surface as the association phase, followed by the injection of running buffer as the dissociation phase. The parameters of the setup were as follows: association contact time of 180 s, dissociation contact time of 540 s, flow rate of 30 µL/min, and sample concentration of 320 nM. All data were processed using Biacore 8 K Evaluation software version 1.1. Flow cell 1 and the blank injection of buffer in each cycle were used as double references for subtraction. The instruments and reagents used were as follows: Biacore 8 K, 29215379–2177839 (GE Healthcare); Series S Sensor Chip CM5, Cat. No. BR-1005–30 (Lot. No. 10261891) (GE Healthcare); HBS-EP, 10 mM HEPES, 150 mM NaCl, 3 mM EDTA, 0.05% Tween 20, pH 7.4, Cat. No. BR-1006–69 (Lot. No. BCBW7263) (GE Healthcare); amine coupling kit, Cat. No. BR-1000–50 (Lot. No. 2085040) (GE Healthcare); NHS, 100 mM N-hydroxysuccinimide in H_2_O; EDC, 400 mM 1-ethyl-3-(3-dimethylaminopropyl) carbodiimide in H_2_O; ethanolamine:1 M ethanolamine hydrochloride, adjusted to pH 8.5 with NaOH; 10 mM sodium acetate, pH 4.5, Cat. No. BR-1003–50 (Lot. No. 20278962371) (GE Healthcare).

The affinities were also compared by ELISA. Briefly, the plates were coated with 100 µL/well 1.5 µg/mL human DSG2 in HBS-N (Ca^2+^) at 4°C overnight, then washed with 0.05% Tween-HBS-N (Ca^2+^). Serially diluted Ad3K, Ad7K, Ad55K, and Ad5K were then added and incubated for 2 h with low-speed shaking. The plates were then incubated with an HRP-labeled anti-His antibody for 45 min. The substrate was added to read at A450.

For knob competition experiments, A549 cells were seeded in 24-well plates and cultured for 24 h. The cells were washed with cold PBS. Serially diluted recombinant knobs were prepared in PBS, added to the cells, and incubated for 1 h on ice with constant shaking. Subsequently, EGFP-expressing adenoviruses were added and incubated for 2 h. After washing twice with cold PBS, the cells were cultured in a fresh medium for 2 days. Cells were photographed using a Leica DMIL DFC3000G fluorescence microscope equipped with LAX V4.10 software, and the number of fluorescent cells was counted.

### Generation of recombinant Ads

The recombinant replication-competent adenovirus, rAd3E, was generated as previously described (44). The recombinant replication-competent adenoviruses, rAd7E and rAd55E, were generated using the similar homologous recombination method from HAdV-7 and HAdV-55 strains, respectively. The plasmid construct pBRAd3-EGFP was used to rescue the recombinant virus rAd3E. Modified rAd3E-K7 with chimeric fiber replaced with the fiber knob from HAdV-7 and rAd3E-K55. Then, modified fiber replaced with the fiber knob from HAdV-55 was generated as previously described (47, 48). Briefly, the shuttle vector pshuttle-A3-fiber was obtained by PCR and ligation. The fiber knob gene from HAdV-7 (K7) or HAdV-55 (K55) replaced the HAdV-3 fiber knob (K3) gene of pshuttle-A3-fiber to construct shuttle vectors pshuttle-Ad7knob or pshuttle-Ad55knob with ClonExpress Entry One Step Cloning Kit (Vazyme, Nanjing, China). Finally, pBRAd3-EGFP and pshuttle-Ad7knob or pshuttle-Ad55knob were digested with *Pme*I and *Rsr*II and ligated to obtain the plasmid pBRAd3-EGFP-Ad7knob or pBRAd3-EGFP-Ad55knob. The constructs were confirmed by restriction digestion and sequencing. Next, pBRAd3-EGFP-Ad7knob and pBRAd3-EGFP-Ad55knob were linearized and transfected into A549 cells using Lipofectamine 3000 (Invitrogen) to rescue rAd3E-K7 and rAd3E-K55, respectively.

The replication-defective adenovirus type 5 expressing EGFP (rAd5E) was rescued with the pAdEASY system and maintained in our lab. Modified replication-defective adenoviruses rAd5E-F3, rAd5E-F7, and rAd5E-F55 were generated by replacing the whole fiber gene of rAd5E with fiber genes from HAdV-3, HAdV-7, and HAdV-55, respectively.

### Plaque-forming assay

A549 cells were seeded in 6-well plates and incubated for 12–24 h to confluency. The cells were then inoculated with 0.5 mL of 10-fold serial dilutions of viral stocks, HAdV-3, HAdV-7, HAdV-55, rAd3E, rAd7E, rAd55E, rAd3E-K7, or rAd3E-K55, and incubated for 2 h at 37°C with rocking every 15 min. The medium was then removed, and the cells were washed with PBS. Equal volumes of 2% low melting point agarose and 2× DMEM supplemented with 4% FBS were mixed and overlaid the cell monolayers to solidify at room temperature. The plates were then incubated at 37°C with 5% CO_2_. An additional overlay was added at 4 and 8 dpi. After incubation for 12 days, overlays were removed, and the cells were fixed and stained with 4% formaldehyde plus 0.5% crystal violet. Plaque size was measured using Visionworks software, and plaque-forming units were calculated in PFU/mL.

### Infection fluorescent foci size assay

293T cells were seeded in 6-well plates and incubated until confluence. The cells were then inoculated with 0.5 mL of 10-fold serial dilutions of rAd5-F3, rAd5-F7, and rAd5-F55 viral stocks and incubated for 2 h at 37°C with rocking every 15 min. The infection medium was then removed, and the cells were washed with PBS. Equal volumes of 2% low melting point agarose and 2× DMEM supplemented with 4% FBS were mixed and overlaid the cell monolayers. The agarose was allowed to solidify at 25°C and incubated at 37°C with 5% CO_2_. At 4 dpi, images were obtained using a Leica DMIL DFC3000G fluorescence microscope equipped with LAX V4.10 software, and infection fluorescent foci size was measured using Visionworks software.

### Comparison of virus infection ability

A549 and 293T cells were seeded in 96-well plates and incubated to approximately 90% confluence. The cells were then infected with one fluorescence-forming units/cell rAd3E, rAd7E, rAd55E, rAd3E-K7, rAd3E-K55, rAd5-F3, rAd5-F7, or rAd5-F55. After 30 or 60 min adsorption, the medium was removed, cells were washed twice with PBS, and fresh medium was added and incubated for 24 h at 37°C in 5% CO_2_. Fluorescence images were taken using a Leica DMIL DFC3000G fluorescence microscope equipped with LAX V4.10 software, and the number of fluorescent cells was counted.

### Virus replication kinetics of genome copies and fluorescence-forming units

A549, HBEpiC, or 293T cells were seeded in 96-well plates and incubated to approximately 90% confluence. The cells were infected with the same genome copies (5.78 × 10^4^ copies/mL) of rAd3E, rAd7E, rAd55E, rAd3E-K7, rAd3E-K55, rAd5-F3, rAd5-F7, and rAd5-F55. After incubation for 4 h, cells were washed with PBS and collected at 4, 12, 24, 48, 72, and 96 h post-infection.

TaKaRa MiniBEST Viral RNA/DNA Extraction Kit (TaKaRa, Dalian, China) was used to extract adenovirus DNA according to the manufacturer’s instructions, which was then quantified using TaqMan real-time PCR kit for HAdVs (Guangzhou HuYanSuo Medical Technology Co., Ltd., Guangzhou, China) as previously reported (18).

The collected viral content was frozen and thawed thrice and centrifuged at 12,000 × *g* for 15 min at 4°C to separate the supernatant. The supernatant was serially diluted 10-fold, and cells were infected for 48 h at 37°C in 5% CO_2_. Fluorescence images of the cells were obtained using a Leica DMIL DFC3000G fluorescence microscope equipped with LAX V4.10 software. The number of fluorescent cells was counted, and FFUs were calculated as described above.

### HAdV infection of differentiated HBEpiC

HBEpiCs were seeded on collagen-coated 0.4-µm pore size transwell inserts (Corning) in 24-well plates at a density of 2 × 10^4^ cells/well with EpiX medium and incubated to approximately 100% confluence. Then the differentiation medium (1:1 Dulbecco’s modified Eagle medium: Nutrient Mixture F-12) containing 2% Ultroser G serum substitute (Pall BioSepra, Cergy-Staint-Christophe, France) was added to the basolateral chamber, and the medium in the apical chamber was removed by aspiration. Every 2 days, the differentiation medium was replaced. Transepithelial electrical resistance values were determined using a Millicell ERS meter (Millipore). When TEER values were >1,000 Ω, the cells were considered well differentiated and could be used for subsequent studies of the model.

Well-differentiated HBEpiCs were infected with 50 FFU of rAd3E, rAd7E, rAd55E, rAd3E-K7, or rAd3E-K55. After incubation for 4 h, the cells were washed two times, and cell fluorescence images were taken using a Leica DMIL DFC3000G fluorescence microscope equipped with LAX V4.10 software at 24, 48, 72, 96, and 120 hpi. The cells were washed and collected at 48 and 96 hpi, and HAdVs in the collection were quantified by qPCR assays, as described above. The viruses in the apical chamber and the basolateral chamber were collected every 2 days, and the FFUs of the viruses were quantified. TEER was measured every 2 days using a Millicell ERS meter (Millipore).

### Animal experiments

Humanized DSG2 KI homozygous C57BL/6 mice, Ho-hDSG2-C57, with human DSG2 cDNA inserted into the DSG2 gene, were generated by Cyagen Biosciences (Suzhou, China) with TALEN (Transcription Activator-Like Effector Nuclease) knock-in technology. Specific pathogen-free wild-type C57BL/6 mice were purchased from Guangdong Medical Laboratory Animal Center. Primary mouse cells were obtained from kidneys and cultured in DMEM and 20% fetal bovine serum. Primary cells were infected with replication-deficient recombinant human adenoviruses, and images were observed using a Leica DMIL DFC3000G fluorescence microscope. Fourteen female Ho-hDSG2-C57 mice were injected intranasally with 100 µL 1 × 10^10^ VPs of live rAd3E, rAd3E-K7, rAd3E-K55 (*n* = 4 per virus group, two males and two females), or PBS (*n* = 2, one male and one female). Mice were humanely euthanized on day 2 after infection, and their lung and liver tissues were collected. Parts of lung tissue were homogenized and analyzed for HAdV genome copies by qPCR, as described above. Tissues were fixed with 10% neutral buffered formalin, embedded in paraffin, and sliced for hematoxylin and eosin and immunohistochemical staining. Tissue sections were stained according to standard procedures (49) and blindly assessed by a pathologist. For immunohistochemical staining, HAdV-3 antigens were detected by anti-HAdV-3 mouse serum as the primary antibody and anti-PBS mouse serum as a negative control.

### Statistical analysis

Statistical analysis and graphing were performed with GraphPad Prism 7.04 software (GraphPad Software, San Diego, USA). Differences between groups were calculated by the Kruskal-Wallis test followed by Dunn’s multiple comparisons test. Data are expressed as the mean ± standard deviation. Significant differences between groups are denoted by *(*P* < 0.05), **(*P* < 0.01), ***(*P* < 0.001), ****(*P* < 0.0001), or not significant (ns) (*P* > 0.05).

## Data Availability

All data generated or analyzed during this study are included in this published article and its supplemental files. The primary data is available from the authors on request.

## SUPPLEMENTAL MATERIAL

Supplemental material spectrum.01090-23-s0001.pdf Fig. S1 to S4.

ASM does not own the copyrights to Supplemental Material that may be linked to, or accessed through, an article. The authors have granted ASM a non-exclusive, world-wide license to publish the Supplemental Material files. Please contact the corresponding author directly for reuse.
