# Supplementary material for: Higher affinities of fibers with cell receptors increase the infection capacity and virulence of human adenovirus type 7 and type 55 compared to type 3
Source: Microbiol Spectr. 2023 Nov 29;12(1):e01090-23. doi: 10.1128/spectrum.01090-23 (PMC10783091; doi:10.1128/spectrum.01090-23)
Supplement: Supplemental material — Fig. S1 to S4. [file spectrum.01090-23-s0001.pdf]

Supplemental data

Figure S1. Infection and replication characterization of recombinant rAd3E, rAd7E and rAd55E. Plaque size distribution **(a)** and plaque formation unit **(b)** of rAd3E, rAd7E and rAd55E. **(c)** A549 cells were infected with rAd3E, rAd7E, and rAd55E for 30 min or 60 min, washed three times, and observed under a fluorescence microscope at 24 h post-infection**. (d)** Fluorescent cell numbers were counted 24 h post-infection. **(e)** Viral genome DNA proliferation curves and **(f)** infectious virus growth kinetics of rAd3E, rAd7E, and rAd55E. Viral genome copy numbers were determined by qPCR. Infectious virus titers were determined by counting fluorescence forming units (FFU). The viruses infected in A549 cells were collected and determined at 4, 12, 24, 48, 72, and 96 h post-infection. Each experiment was repeated independently three times, and the mean values and standard deviations are shown. Statistical analysis was performed using the Kruskal-Wallis test followed by Dunn's multiple comparisons test. ***$P < 0.001$; **$P < 0.01$; *$P < 0.05$.

Figure S2. Infection characterization of rAdV3E, rAdV7E, rAdV55E, rAdV3E-K7, and rAdV3E-K55 in differentiated HBEpiC. Differentiated HBEpiC cells infected with recombinant HAdVs were observed under a fluorescence microscope.

Figure S3. Virus replication and TEER detection of differentiated HBEpiC infected by rAdV3E, rAdV7E, rAdV55E, rAdV3E-K7, and rAdV3E-K55. **(a and b)** Viral copies (FFU) in the basolateral chambers of well-differentiated HBEpiC model infected with rAdV3E, rAdV7E, rAdV55E, rAdV3E-K7, and rAdV3E-K55 at 0, 2, 4, 6, 8, 10, and 12 days post-infection. **(c and d)** TEERs of well-differentiated HBEpiC model infected

with recombinant HAdVs measured every 2 days . Each experiment was repeated independently three times, and the mean values and standard deviations are shown.

Figure S4. Infection characterization of EGFP-expression rAd5-F3, rAd5-F7, and rAd5-F55 in primary kidney cells from DSG2 humanized mouse Ho-hDSG2-C57 and wild C57 mouse. (a) Primary kidney cells were isolated from mice and cultured in 24-well plates. Cells were then infected with EGFP-expression replication-deficient HAdVs. After 48 h, fluorescent cells were observed and counted under a fluorescence microscope. (b) Primary kidney cells from Ho-hDSG2-C57 infected with rAd5-F7, rAd5-F55 and rAd5-F3 under a fluorescence microscope. Statistical analysis was performed using the Kruskal-Wallis test followed by Dunn’s multiple comparisons test. ****$P < 0.0001$.

Fig. S1

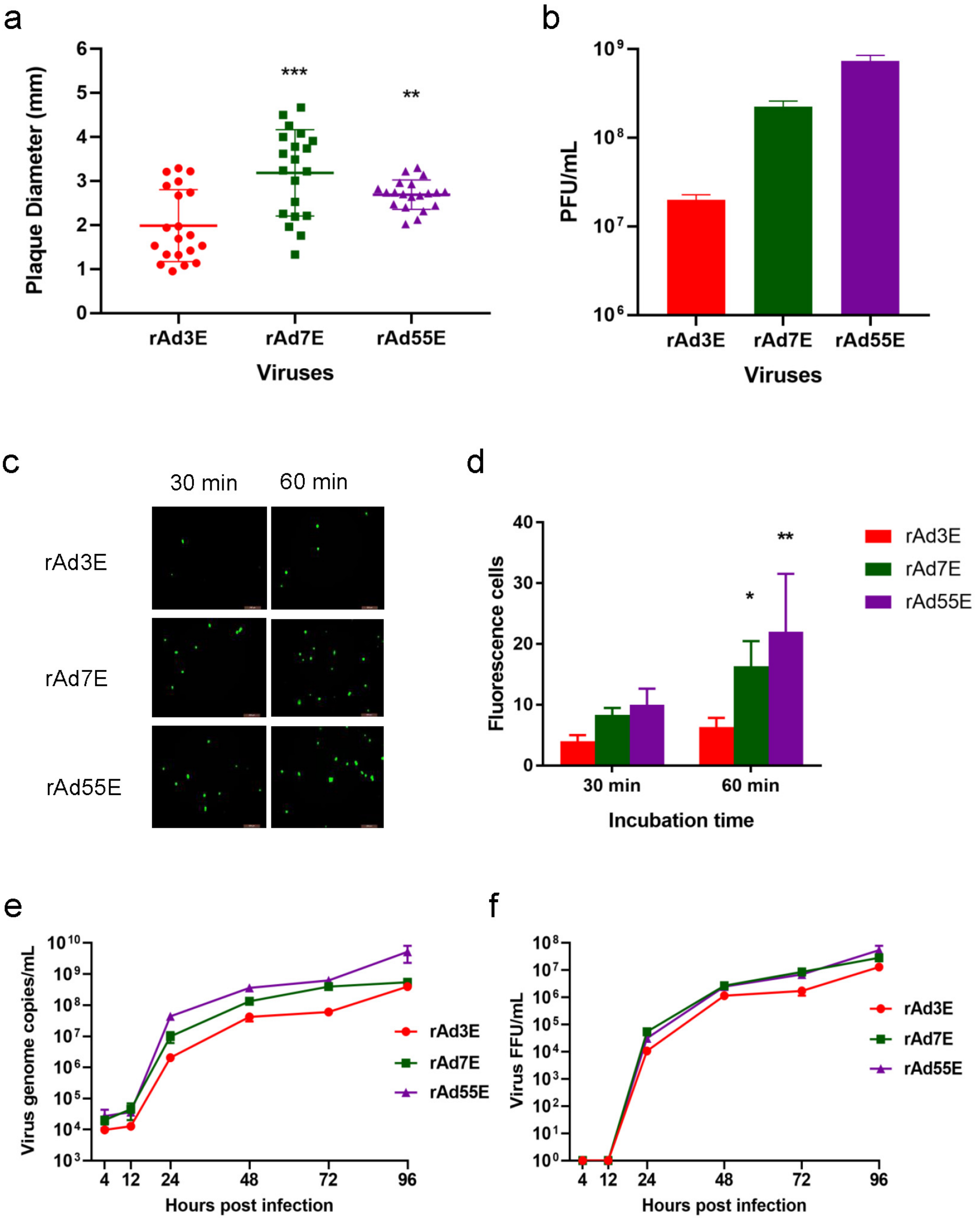

a
Plaque Diameter (mm)
0
1
2
3
4
5
6
***
**
rAd3E
rAd7E
rAd55E
Viruses
b
PFU/mL
10^9
10^8
10^7
10^6
rAd3E
rAd7E
rAd55E
Viruses
c
30 min
60 min
rAd3E
rAd7E
rAd55E
d
Fluorescence cells
0
10
20
30
40
*
**
30 min
60 min
Incubation time
rAd3E
rAd7E
rAd55E
e
Virus genome copies/mL
10^{10}
10^9
10^8
10^7
10^6
10^5
10^4
10^3
4
12
24
48
72
96
Hours post infection
rAd3E
rAd7E
rAd55E
f
Virus FFU/mL
10^8
10^7
10^6
10^5
10^4
10^3
10^2
10^1
10^0
4
12
24
48
72
96
Hours post infection
rAd3E
rAd7E
rAd55E

Fig. S2

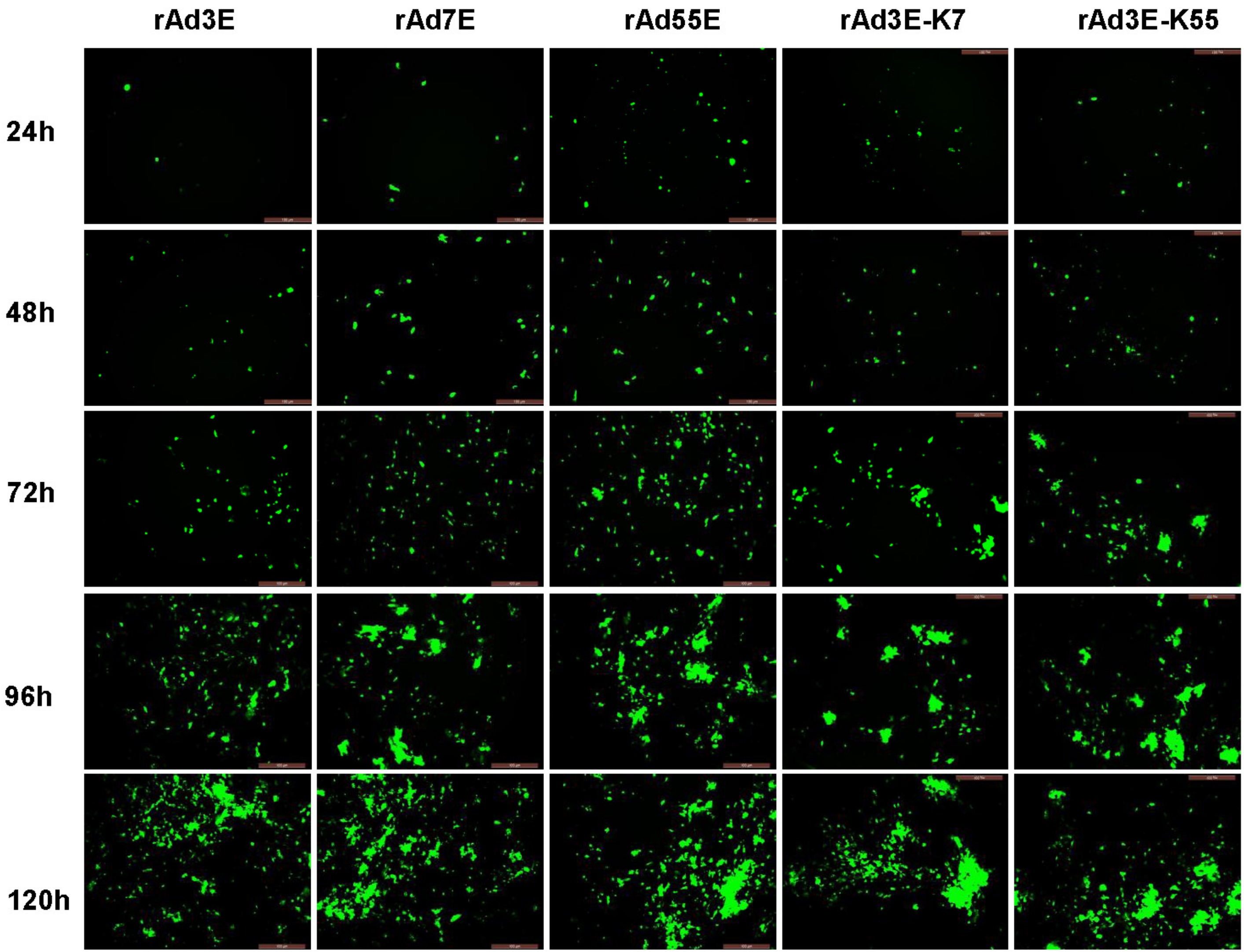

Fig.S3

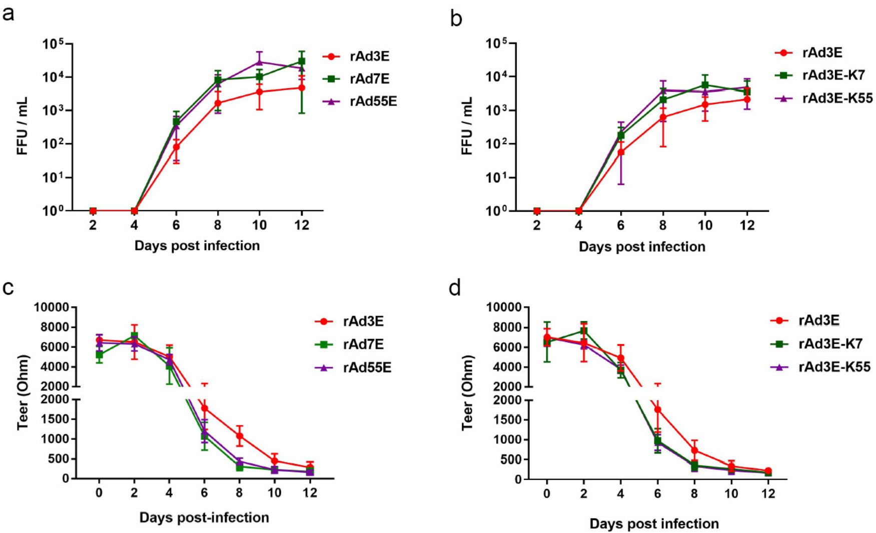
a
FFU / mL
Days post infection
rAd3E
rAd7E
rAd55E
b
FFU / mL
Days post infection
rAd3E
rAd3E-K7
rAd3E-K55
c
Teer (Ohm)
Days post-infection
rAd3E
rAd7E
rAd55E
d
Teer (Ohm)
Days post infection
rAd3E
rAd3E-K7
rAd3E-K55

Fig.S4

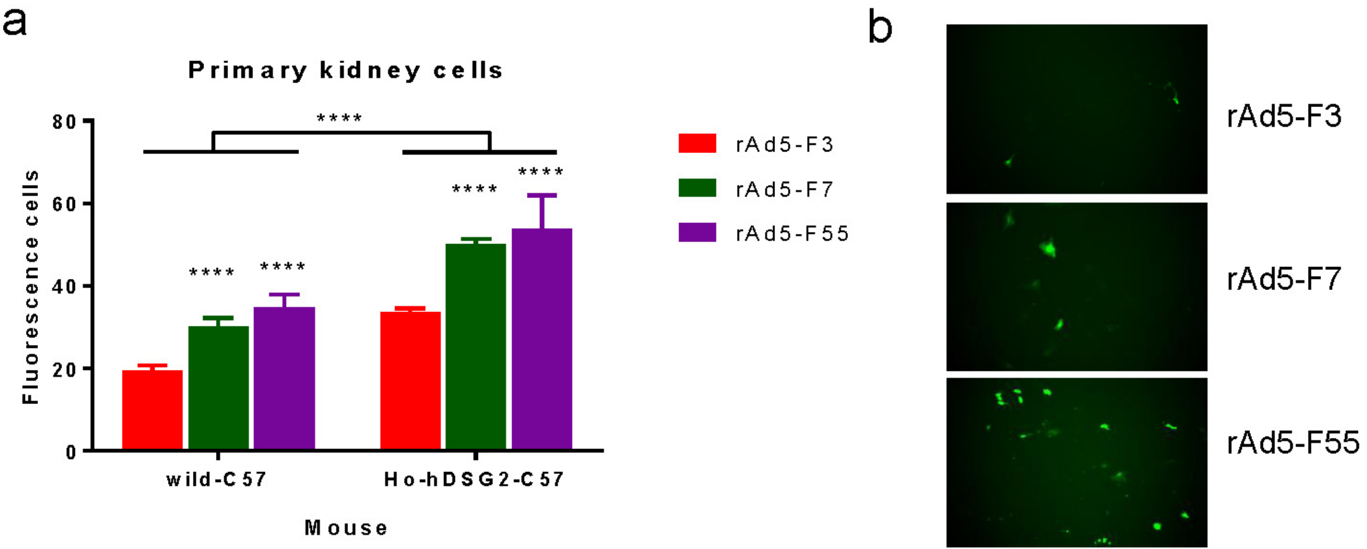
a
Primary kidney cells
80
60
40
20
0
Fluorescence cells
****
****
****
****
****
wild-C57
Ho-hDSG2-C57
Mouse
rAd5-F3
rAd5-F7
rAd5-F55
b
rAd5-F3
rAd5-F7
rAd5-F55
